# Up-Regulation of SALL4 Is Associated With Survival and Progression *via* Putative WNT Pathway in Gastric Cancer

**DOI:** 10.3389/fcell.2021.600344

**Published:** 2021-02-11

**Authors:** Yang Yang, Xiaohong Wang, Yiqiang Liu, Ying Hu, Zhongwu Li, Ziyu Li, Zhaode Bu, Xiaojiang Wu, Lianhai Zhang, Jiafu Ji

**Affiliations:** ^1^Key Laboratory of Carcinogenesis and Translational Research (Ministry of Education), Department of Tissue Bank, Peking University Cancer Hospital and Institute, Beijing, China; ^2^Department of Pathology, Peking University Cancer Hospital and Institute, Beijing, China; ^3^Gastrointestinal Cancer Center, Peking University Cancer Hospital and Institute, Beijing, China

**Keywords:** gastric cancer, SALL4, immunohistochemistry, prognosis, weighted gene co-expression network analysis, enrich pathway analysis, Wnt signaling pathway

## Abstract

*SALL4*, a transcriptional factor involved in embryonic stem cell self-renewal and pluripotency, is overexpressed in gastric cancer (GC). However, the association of *SALL4* with the survival of GC patients is not well-understood, and the role of *SALL4* in cancer progression is still unknown. In the present study, a total of 1,815 GC patients who underwent radical resection at Peking Cancer Hospital were included consecutively from 2015 to 2018, confirming the prognostic value of SALL4 and validating by data from TCGA and GEO. The protein and mRNA expression levels of SALL4 were evaluated by immunohistochemistry and qPCR, respectively. Besides, GSEA and WGCNA were applied to explore the SALL4-related cancer-promoting signaling pathways and gene modules. Our results showed that overexpression of SALL4 was observed in 16.7% of GC patients. SALL4 positivity was associated with male, older age, mixed-type histology, late stages, lymphatic metastasis, vascular invasion, non-cardia location, high AFP level, and no EBV infection background. *SALL4* could be served as a marker for prognostic prediction in GC, and SALL4-positive GC was significantly associated with shortened survival. Further, the bioinformatic analysis indicated that the Wnt/β-catenin signaling pathway was activated in *SALL4*-high cases compared with *SALL4*-low cases. Expression of *SALL4* was also positively correlated with the expression of multiple co-expressed genes, such as *TRIB3*, which plays an important role in activating the Wnt/β-catenin pathway. Our findings indicate that *SALL4* is associated with clinicopathological features related to cancer progression in GC and its function in the Wnt/β-catenin pathway.

## Introduction

Gastric cancer (GC) is the third leading cause of cancer-related mortality worldwide (Bray et al., 2018). The incidence and mortality rates for patients with GC have declined recently. However, despite recent advancements in treatment strategies, the prognosis of patients with GC is still poor, and the 5-year survival rate is <30% (Araki et al., 2018; Cats et al., 2018; Kudou et al., 2018). Therefore, the identification of novel biomarkers related to GC progression is imperative. The ideal markers not only would promote the current understanding of GC pathogenesis but also could reveal new effective strategies for GC treatment to us.

Spalt-like transcription factor 4 (*SALL4*), located on chromosome 20q13.2, encodes a zinc finger transcription factor that plays a key role in maintaining the pluripotency and self-renewal capacity of embryonic stem cells (Zhang et al., 2014). SALL4 was first described in human acute myeloid leukemia (Ma et al., 2006). Subsequently, overexpression of SALL4 was reported in a variety of cancers, such as breast cancer, lung cancer, liver cancer, endometrial cancer, germ cell cancer, as well as GC (Camparo and Comperat, 2013; Gonzalez-Roibon et al., 2013; Li et al., 2015; Zhang et al., 2015; Tatetsu et al., 2016). Numerous studies have revealed the role of SALL4 in the process of carcinogenesis, including invasion and metastasis, cell proliferation, stemness, and apoptosis (Liu et al., 2015; Kim et al., 2017).

Previous research conducted at the mRNA level has suggested that SALL4 is a factor for poor prognosis in GC (Zhang et al., 2014; Yanagihara et al., 2015). However, the association between SALL4 protein expression and the clinical outcome in patients with GC is not yet defined and the functional role of SALL4 in GC is still unknown. This study aimed to comprehensively analyze SALL4 expression in GC and explore its prognostic value, along with the underlying mechanism. We determined SALL4 protein expression in GC and analyzed its relationship with clinicopathological features. Then, using The Cancer Genome Atlas (TCGA) data, we explored the regulation of *SALL4* gene expression by analyzing the correlation between DNA copy number variation (CNV) and aberrant *SALL4* mRNA expression in GC. Besides, bioinformatics analysis was used to investigate the relevant pathways and co-expression genes of *SALL4* in GC, including gene set enrichment analysis (GSEA), weighted gene co-expression network analysis (WGCNA), and differential expression gene analysis, which provides new insight into the follow-up research.

## Materials and Methods

### Patients

GC patients, who underwent radical resection at Peking University Cancer Hospital between 2015 and 2018, were consecutively enrolled. Patients without paraffin-embedded clinical tissue specimens or incomplete clinicopathological information were excluded. In total, 1,815 cases were eligible for analyses. TNM stage was determined in accordance with the 7th edition of classification recommended by the American Joint Committee on Cancer (AJCC). All patients underwent follow-up evaluations until October 2020. This investigation was approved by the Ethics Committee of Peking University Cancer Hospital. Informed consent was obtained from each patient at the time of sample collection.

### Immunohistochemistry Staining

Four-micrometer-thick sections of formalin-fixed paraffin-embedded tissues were mounted on poly-L-lysine-coated slides. Then, the slides were deparaffinized in xylene and rehydrated with a gradient of ethanol and distilled water. Endogenous peroxidase activity was quenched with 3% hydrogen peroxide for 10 min at room temperature. The slides were inactivated by incubation in 10 mmol/L ethylenediaminetetraacetic acid (EDTA; pH 8.0) for 3 min. The sections were incubated overnight at 4°C with mouse anti-SALL4 antibody (1:100) (Zsbio; Beijing, China). The primary antibodies were probed with a two-step Poly-HRP Anti-Mouse/Rabbit IgG Detection System (Zsbio; Beijing, China). Positive and negative controls in immunohistochemistry (IHC) were routinely used. For the negative controls, the primary antibody was replaced with non-immune mouse serum to confirm specificity. We also used an internal positive control (seminoma tissue) for quality assurance.

### Quantitative RT-PCR Analysis

Reverse transcription-quantitative real-time PCR (RT-qPCR) was performed to determine the relative *SALL4* mRNA expression levels in the clinical samples as previously described (Wang et al., 2017). Extracted mRNA was amplified with human *SALL4*-specific primers by q-PCR. The human *GAPDH* was included as an internal control. Each sample was assessed in triplicate. Expression of gene was given as the ratio of expression of target gene mRNA to that of *GAPDH* mRNA.

### Pathological Scoring

Evaluation of SALL4 staining was principally based on the scoring criteria described previously (Yong et al., 2013). Briefly, only nuclear reactivity with a diffuse pattern was considered as SALL4 positive. Patchy granular nuclear reactivity was scored as negative (Liu T.-C. et al., 2014). SALL4 expression was classified according to a semi-quantitative score based on the percentage of tumor cells displaying a diffuse nuclear pattern of SALL4 immunoreactivity, as described previously: 0, <5%; 1, 5–30%; 2, 31–50%; 3, 51–80%; 4, >80%. Scores >1 were defined as positive (Cao et al., 2009b; Yong et al., 2013). SALL4 expression in GC tissues was scored independently by two senior pathologists. If any disagreement arose during the evaluation, a third pathologist was consulted.

### Retrieval of TCGA and Gene Expression Omnibus Data

Gene expression profiles and clinical and survival data of GC patients in the TCGA stomach adenocarcinoma (STAD) dataset (*n* = 407) were downloaded from the TCGA Data Portal (https://portal.gdc.cancer.gov/). The survival data and microarray datasets of GSE15459, GSE34942, GSE57303, and GSE62254 were obtained from the gene expression omnibus (GEO) database (https://www.ncbi.nlm.nih.gov/geo/), which included a total of 626 Asian GC patients. After array annotations, all samples in four datasets were integrated to reduce deviation and variability by batch normalization. Also, *SALL4* mRNA expression and copy number data were obtained from the UCSC Xena browser (http://xena.ucsc.edu/).

### Gene Set Enrichment Analysis

Gene set enrichment analysis (GSEA) was used to further elucidate the significant enriched pathway data between high and low *SALL4* expression group. Transcription data from four GEO and TCGA datasets were analyzed by GSEA 3.0 software (Broad Institute; Cambridge, Massachusetts, USA), and the number of permutations was set to 1,000. The mean expression value of each gene was used for correlation analysis.

### Screening for SALL4-Related Gene Modules by Weighted Gene Co-Expression Network Analysis

Based on GEO microarray data (GSE15459, GSE34942, GSE57303, and GSE62254), *SALL4*-related gene modules were identified using WGCNA. Correlation coefficients between the module eigengenes and traits were calculated by using Pearson's approach. High *SALL4*-related gene modules were defined as those with maximum correlation coefficients. We then constructed a PPI network by the STRING database using Cytoscape (V.3.8.0) for network visualization.

### Functional Enrichment and Differentially Expressed Genes Analysis of Co-Expression Modules

The pathway enrichment analysis was performed on these genes in the most relevant gene modules using Enrichr (https://amp.pharm.mssm.edu/Enrichr/). The DEGs of high *SALL4* expression-related gene modules were identified using the R package limma with a threshold of |log2FoldChange| >1 and *P* < 0.05.

### Statistical Analysis

Statistical analysis was performed using SPSS software version 22.0 (IBM, Armonk, NY, USA). The association between SALL4 expression and clinicopathological parameters in GC was assessed by the chi-square test. Univariate analysis of cumulative overall survival was conducted using a Cox regression model. Overall survival and its association with SALL4 protein expression were evaluated by the Kaplan**–**Meier method followed by the log-rank test. Subsequent multivariate analysis of prognostic factors was conducted using the Cox regression model. Unpaired Student's *t*-test and a receiver operating characteristic (ROC) curve were performed to assess the differential expression of *SALL4* in GC and normal tissues. The correlation between copy number and *SALL4* mRNA expression was also analyzed. *SALL4* mRNA expression and co-expressed genes were evaluated by linear regression and correlation analyses. The significance threshold is *P* < 0.05.

## Results

### Patient Characteristics

A total of 1,815 consecutive patients with GC (1,296 males and 519 females) were included in this study. The mean age at diagnosis was 59 ± 10 years. Most cancers were diagnosed at relatively early stages (27.7% at stage I and 29.6% at stage II), localized to the non-cardia sites of the stomach (75.6%), and classified as Lauren intestinal type (38.3%). Of 1,812 patients for whom lymph node status was available, 1,072 (59.1%) had lymph node metastasis. Vascular invasion was observed in 974 (53.7%) out of 1,811 patients (Supplementary Table 1).

### Expression of SALL4 in GC and Its Correlation With Clinicopathological Features

**Figure 1:**
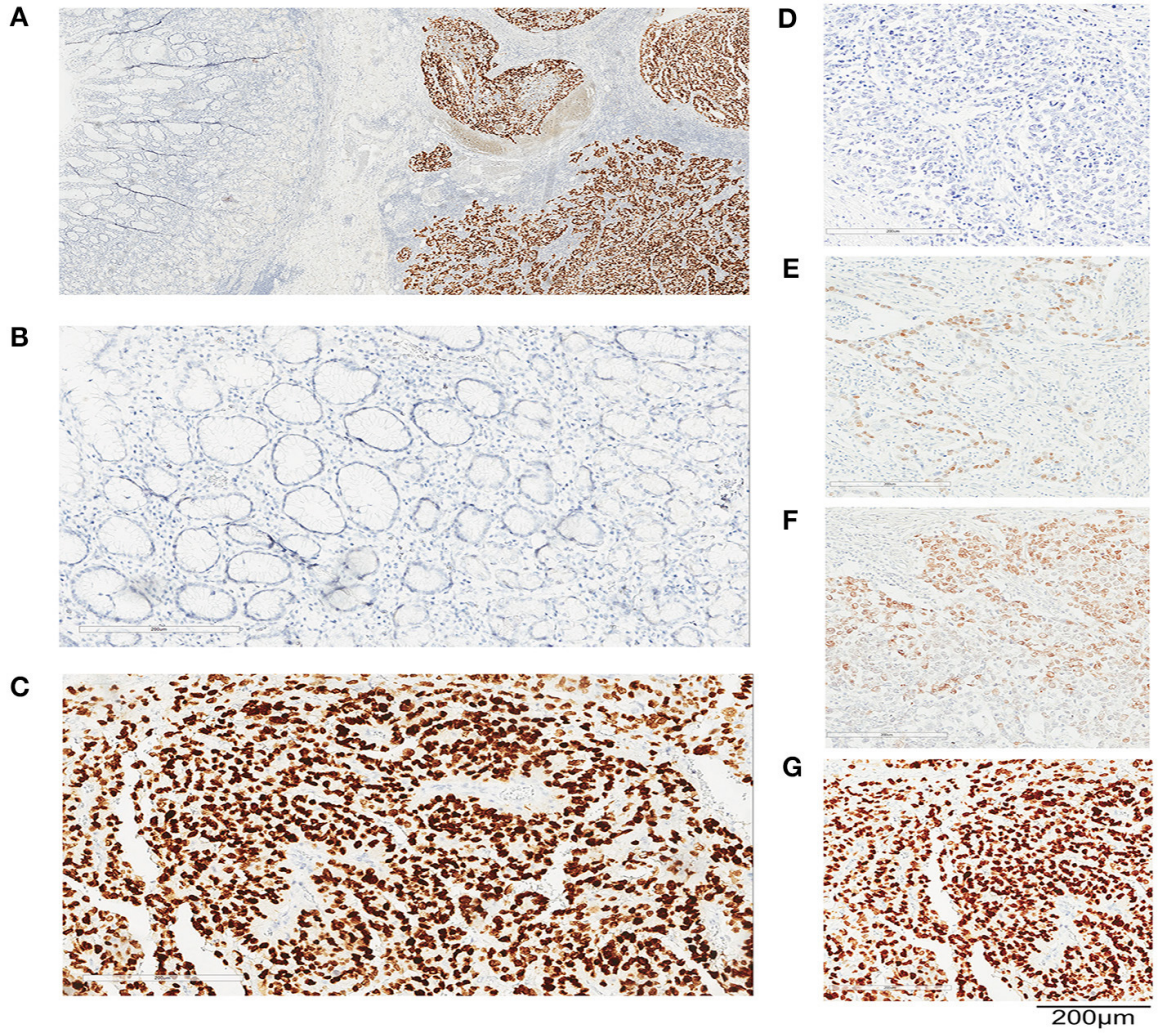
SALL4 expression in human primary gastric cancer (GC) by immunohistochemistry (IHC). Representative images of immunohistochemical staining for SALL4 in GC. Original magnification (×100) **(A)**, adjacent non-tumors gastric mucosa (×200) **(B)**, and cancer (×200) **(C)**. Staining intensity in GC is classified as negative staining **(D)**, weak staining **(E)**, moderate staining **(F)**, and strong staining **(G)**. Original magnification, ×200. Bar = 200 μm.

SALL4 staining was localized to the nucleus of cancer cells, and only diffuse nuclear staining was considered positive for SALL4 (Figures 1A–G). Three hundred four (16.7%) tumors were SALL4-positive by IHC. There was rare SALL4 staining in adjacent non-neoplastic tissues (Figures 1A,B). The results of RT-qPCR were concordant with the IHC findings in this sample set (Supplementary Figure 1). Among SALL4-positive and SALL4-negative GCs according to the IHC assessment, the samples were dichotomized into high and low expression subgroups based on RT-qPCR analysis of *SALL4* mRNA (*R* = 0.670, *P* < 0.001).

We observed that the SALL4-positive rate was higher in patients with advanced stage GC (*P* = 0.006), lymph node metastasis (*P* = 0.002), non-cardia localization (*P* = 0.043), and vascular invasion (*P* = 0.023). Tumor SALL4-positive was also more common in Lauren mixed-type GC (*P* < 0.001). In addition, SALL4 expression in tumor cells was more frequent in male patients (*P* = 0.001) and in older patients (*P* = 0.012).

Among the GC subtypes, SALL4 expression was negatively correlated with Epstein–Barr virus (EBV) infection (*P* < 0.001), which is also referred to as EBV associated gastric cancer (EBVaGC) and is generally acknowledged to have a favorable prognosis. In addition, another GC subtype, lymphoepithelioma-like carcinoma (LELC) of GC, featured with intense lymphocytic infiltration and partially overlapped with EBVaGC subtype (Yang et al., 2019), was SALL4-negative. SALL4 was frequently expressed in GC patients with high AFP levels (Table 1).

**Table 1:** Clinicopathological and molecular features according to SALL4 expression.

**Variables**	**SALL4-positive**	**SALL4-negative**	***P*-value**
	**(*n* = 304)**	**(*n* = 1,511)**	
**Sex**			0.001
Male	241	1,055	
Female	63	456	
**Age (years)**			0.012
18–55	89	557	
56–90	215	954	
**Lauren type**			<0.001
Diffuse type	38	467	
Mixed type	155	539	
Intestinal type	111	505	
**Location**			0.043
Cardia	88	355	
Non-cardia	216	1,156	
**TNM stage**			0.006
Stage I	63	439	
Stage II	102	436	
Stage III	139	622	
Stage IV	0	14	
**Lymphatic metastasis**			0.002
Positive	204	868	
Negative	99	641	
Vascular invasion^*^			0.023
Positive	181	793	
Negative	122	715	
Perineural invasion^*^			0.977
Positive	158	786	
Negative	144	719	
**Distant metastasis**			0.092
Positive	0	14	
Negative	304	1,497	
**EBER**			<0.001
Negative	303	1,420	
Positive	1	91	
**WHO classification**			0.044
Conventional adenocarcinoma	304	1,491	
LELC	0	20	
AFP	2.89 ± 2.20	3.33 ± 3.71	0.009

* *Data for Lymphatic metastasis, vascular invasion, and perineural invasion were missing for three, four, and eight patients, respectively; EBER, Epstein–Barr virus-encoded small RNA; LELC, lymphoepithelioma-like carcinoma; WHO, World Health Organization; AFP (ng/L)*.

### Prognostic Significance

Univariate Cox regression analysis revealed that the TNM stage, Lauren type, degree of differentiation, SALL4 expression, lymph node metastasis, vascular invasion, perineural invasion, location, sex, and distant metastasis were the significant prognostic indicators of GC (*P* < 0.05, respectively) (Table 2). In the multivariate model, TNM stage, location, vascular invasion, sex, and the degree of differentiation were statistically significant predictors of mortality. In addition, SALL4-positive predicted poorer survival (HR = 2.211, 95% CI, 1.384–3.533, *P* = 0.001, Table 3).

**Table 2:** Univariate Cox regression analysis of potential poor prognostic factors for gastric cancer patients in our study.

**Variables**	HR^*^ (95% confidence interval)	***P*-value**
**Age (years)**		
18–55	1	
56–90	1.235 (0.830–1.836)	0.297
**Sex**		
Male	0.644 (0.440–0.943)	0.024
Female	1	
**SALL4**		
Positive	2.507 (1.605–3.918)	<0.001
Negative	1	
**Lauren type**		
Diffused type	1.246 (0.957–1.621)	0.102
Intestinal type	0.674 (0.487–0.861)	0.003
Mixed type	1	
**Differentiation**		
Poor	1	
Well or modest	0.499 (0.342–0.729)	<0.001
**Location**		
Cardia	1	
Non-cardia	0.643 (0.431–0.958)	0.030
**TNM stage**		
Stage I	1	
Stage II	6.027 (2.084–17.430)	0.001
Stage III	18.151 (6.651–49.537)	<0.001
Stage IV	36.798 (6.722–201.443)	<0.001
**Vascular invasion**		
Positive	4.700 (2.867–7.704)	<0.001
Negative	1	
**Perineural invasion**		
Positive	2.262 (1.519–3.368)	<0.001
Negative	1	
**Lymphatic metastasis**		
Positive	4.998 (2.941–8.492)	<0.001
Negative	1	
**Distant metastasis**		
Positive	4.036 (0.995–16.374)	0.051
Negative	1	

* *HR, hazard ratio*.

**Table 3:** Multivariate Cox regression analysis of potential poor prognostic factors for gastric cancer patients in our study.

**Variables**	HR^*^ (95% confidence interval)	***P*-value**
**TNM stage**		
Stage I	1	
Stage II	4.079 (1.382–12.045)	0.011
Stage III	9.325 (3.238–26.854)	<0.001
Stage IV	20.033 (3.533–113.577)	0.001
**SALL4**		
Negative	1	
Positive	2.211 (1.384–3.533)	0.001
**Location**		
Cardia	1	
Non-cardia	0.560 (0.371–0.848)	0.006
**Differentiation**		
Poor	1	
Well or modest	0.620 (0.415–0.924)	0.019
**Vascular invasion**		
Positive	1	
Negative	2.107 (1.219–3.643)	0.008
**Sex**		
Female	1	
Male	0.614 (0.411–0.916)	0.017

* *HR, hazard ratio*.

**Figure 2:**
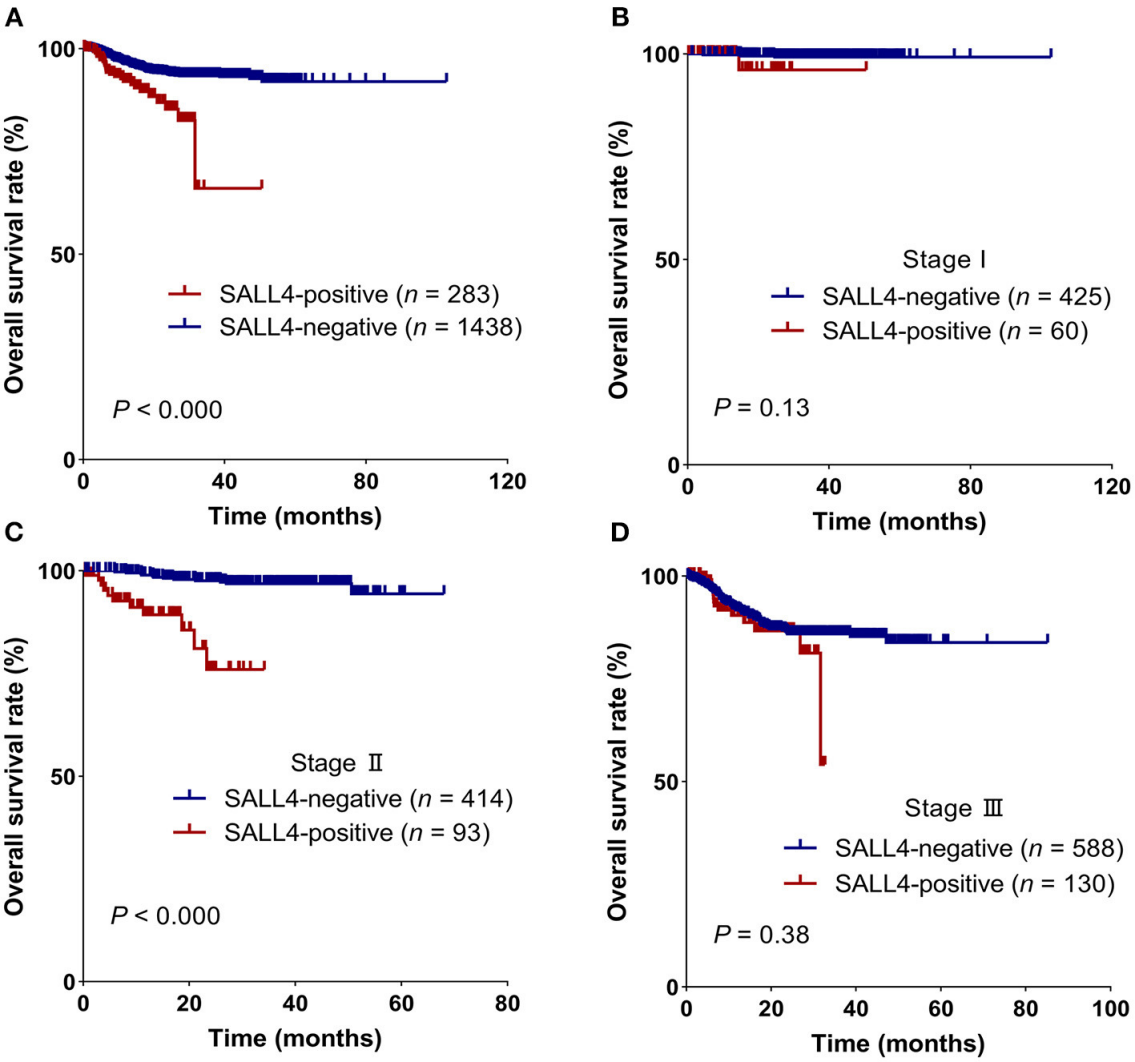
SALL4 is a poor prognostic factor in patients with gastric cancer (GC). Kaplan–Meier survival curves for all patients **(A)**, stage I **(B)**, stage II **(C)**, and stage III **(D)** in different expression groups, which were stratified by SALL4. SALL4-positive is associated with worse outcome in the subgroup of TNM stage II **(C)** patients.

Patients with GC having SALL4-positive expression showed worse survival in the Kaplan**–**Meier survival analysis (3-year overall survival, 0.66 vs. 0.93, log-rank test, *P* < 0.001, Figure 2A). In further analysis, the discrepancy in survival remained significant when patients were stratified by the TNM stage (Figures 2B–D). In stage II GC, patients with SALL4-positive tumors had shorter overall survival than those with SALL4-negative tumors (log-rank test, *P* < 0.001, Figure 2C).

### Analysis of *SALL4* Expression From TCGA Dataset

**Figure 3:**
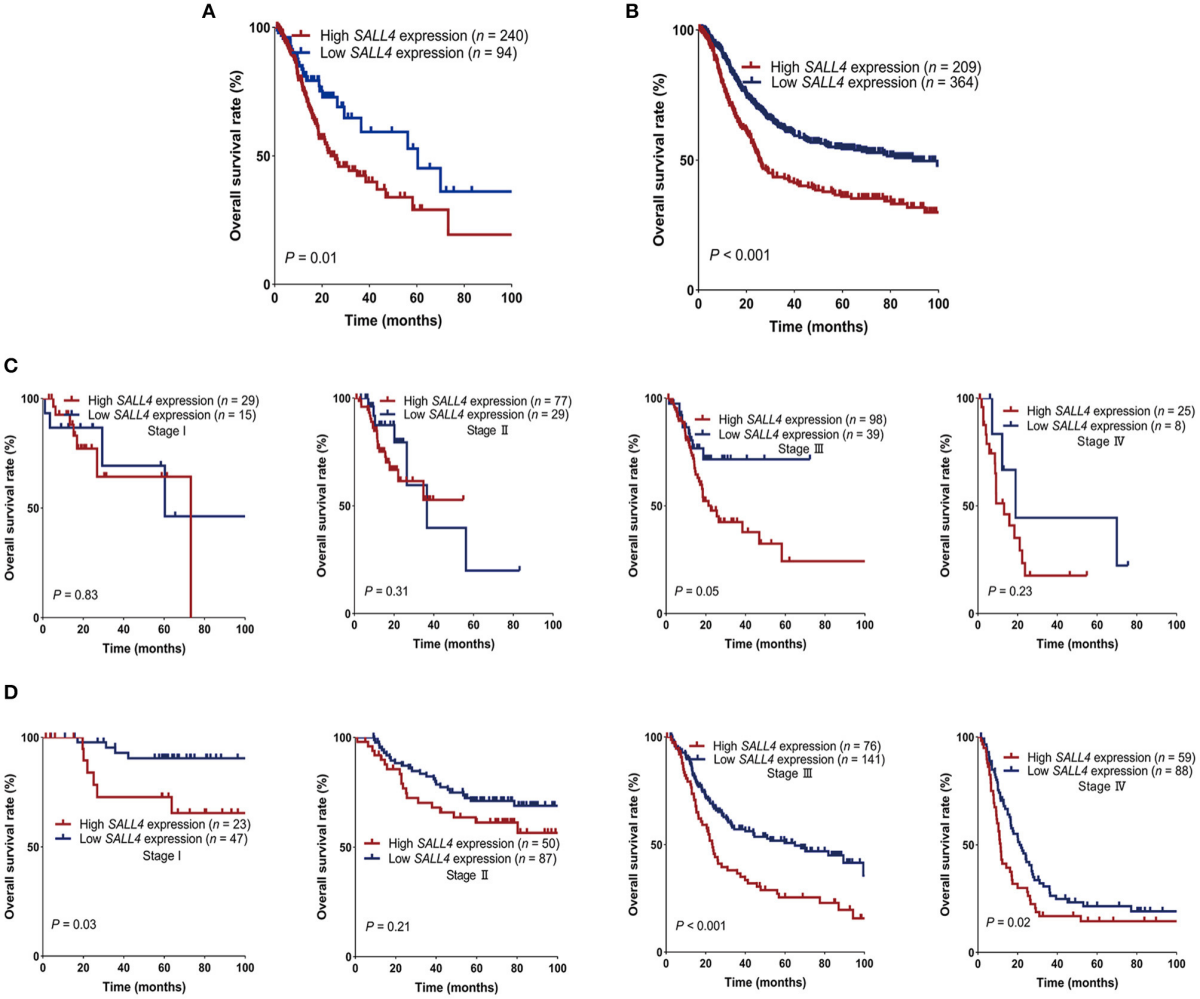
Survival of gastric cancer (GC) patients in The Cancer Genome Atlas (TCGA) and gene expression omnibus (GEO) stratified by *SALL4*. GC patients with high *SALL4* expression had worse prognosis than those with low *SALL4* expression in TCGA **(A)** and GEO **(B)**. Kaplan–Meier survival curves for all stage I, II, III, and IV in different expression groups of TCGA **(C)** and GEO **(D)**. *SALL4* low expression is associated with better outcome in the subgroup of TNM stage III patients.

**Figure 4:**
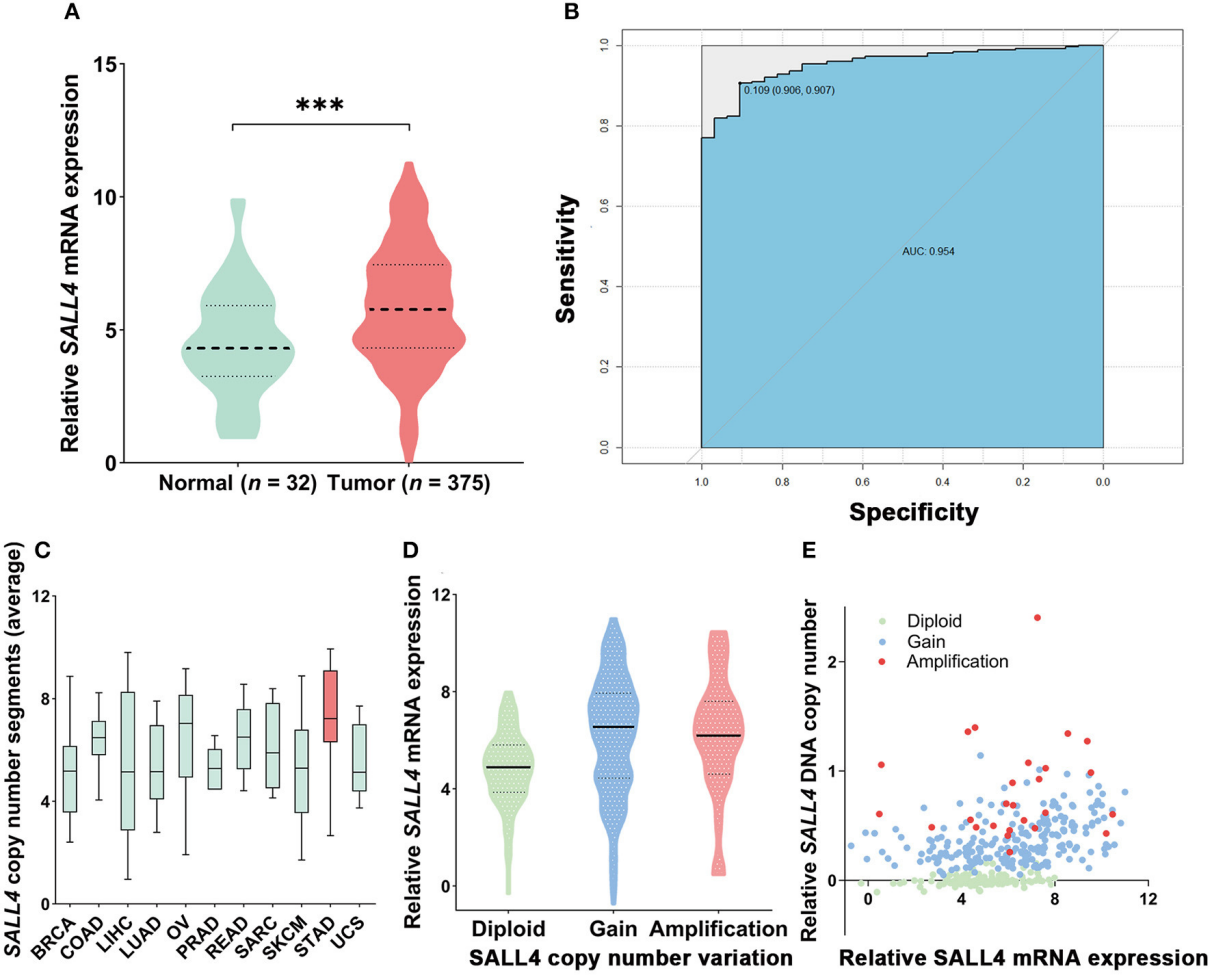
mRNA expression and copy number variant of SALL4 in gastric cancer (GC) in The Cancer Genome Atlas (TCGA). *SALL4* overexpressed in tumor compared to adjacent non-tumor gastric mucosa (***P < 0.001) **(A)**. ROC curve for *SALL4* expression in normal gastric tissue and GC **(B)**. Copy number variant of SALL4 in gastric cancer (GC). A pan-cancer analysis of *SALL4* comparing GC (red) with data from TCGA for other cancer types (green). SALL4 is amplification in various types of cancers, especially GC **(C)**. DNA copy number gain **(D)** is associated with *SALL4* mRNA expression **(E)** in primary GC (BRCA, breast cancer; COAD, colon adenocarcinoma; LIHC, liver hepatocellular carcinoma; LUAD, lung adenocarcinoma; OV, ovarian cancer; PRAD, prostate adenocarcinoma; READ, rectum adenocarcinoma; SARC, sarcoma; SKCM, skin cutaneous melanoma; STAD, stomach adenocarcinoma; UCS, uterine carcinosarcoma).

To compare with our findings, we analyzed the association between *SALL4* mRNA expression and prognosis in the TCGA dataset, which includes 375 GC patients (Supplementary Table 2). *SALL4* mRNA expression was significantly increased in primary gastric tumors when compared with adjacent non-tumor tissues (*P* < 0.001, Figure 4A). We also plotted the area under the ROC curves (AUCs), which illustrated strong separation between the tumor and normal tissues, with an AUC of 0.954 for *SALL4* (Figure 4B). We divided GC patients into two groups (high-expression group vs. low-expression group) according to the analysis of X-title. As shown in the Kaplan**–**Meier survival curve, patients in the high-expression group had shorter survival than those with low or silenced *SALL4* expression (3-year overall survival, 0.42 vs. 0.65, log-rank test, *P* = 0.01, Figure 3A). After being stratified by tumor stage, *SALL4* overexpression predicted poor prognosis in stage III GC patients (*P* = 0.05, Figure 3C), but not in patients with other stages of GC. Note that results at the mRNA level were consistent with our observations at the protein level (Tables 4, 5).

**Table 4:** Univariate Cox regression analysis of prognostic factors for gastric cancer patients in TCGA^*^.

**Variables**	**HR (95% confidence interval)**	***P*-value**
**Age (years)**		
18–55	1	
56–90	1.793 (1.027–3.128)	0.040
**Sex**		
Male	1.333 (0.904–1.965)	0.147
Female	1	
**SALL4**		
High	1.724 (1.115–2.666)	0.014
Low	1	
**Differentiation**		
Poor	1	
Well or modest	0.784 (0.541–1.136)	0.199
**TNM stage**		
Stage I	1	
Stage II	1.460 (0.725–2.940)	0.290
Stage III	1.935 (1.002–3.699)	0.049
Stage IV	3.779 (1.820–7.850)	<0.001
**Lymphatic metastasis**		
Positive	1.540 (1.002–2.366)	0.049
Negative	1	
**Distant metastasis**		
Positive	2.167 (1.190–3.947)	0.011
Negative	1	

* *TCGA, The Cancer Genome Atlas; HR, hazard ratio*.

**Table 5:** Multivariate Cox regression analysis of potential poor prognostic factors for gastric cancer patients in TCGA^*^.

**Variables**	**HR (95% confidence interval)**	***P*-value**
***SALL4***		
Low	1	
High	1.729 (1.082–2.763)	0.022
**TNM stage**		
Stage I	1	
Stage II	1.686 (0.750–3.789)	0.206
Stage III	2.267 (0.881–5.834)	0.090
Stage IV	4.830 (1.603–14.551)	0.005
**Lymphatic metastasis**		
Positive	0.848 (0.439–1.638)	0.623
Negative	1	
**Distant metastasis**		
Positive	1.037 (0.426–2.526)	0.937
Negative	1	
**Age (years)**		
18–55	1	
56–90	1.691 (1.082–2.763)	0.078

* *TCGA, The Cancer Genome Atlas; HR, hazard ratio*.

### Analysis of *SALL4* Expression in the GEO Dataset

Since the TCGA dataset includes only a few Asian patients with GC, we downloaded raw data and platform information of four datasets of Asian GC patients (GSE15459, GSE34942, GSE57303, and GSE62254) from the GEO database. To lessen the bias of false-positive findings, four datasets were batch corrected and merged, and raw and normalized data are shown in Supplementary Figure 3. Then, the survival data of 573 GC samples from these four datasets were used to analyze the association between *SALL4* mRNA expression and outcomes among Asian GC patients.

Kaplan**–**Meier survival analysis also indicated *SALL4* to be highly correlated with survival (3-year overall survival, 0.43 vs. 0.61, log-rank test, *P* < 0.001, Figure 3B). *SALL4* overexpression predicted poor prognosis in patients with stage I (*P* = 0.02), stage III (*P* < 0.001), and stage IV GC (*P* = 0.02, Figure 3D).

### DNA Copy Number Gain Contributes to the Overexpression of *SALL4* in Primary GC

We also analyzed the mutation level of SALL4 base on the TCGA dataset. Although *SALL4* is rarely mutated (0.1%) in GC, analysis of the TCGA pan-cancer dataset revealed that *SALL4* is amplified in GC and other human cancers, including lung squamous cell carcinoma, colon carcinoma, bladder carcinoma, and lung adenocarcinoma (Figure 4C). A positive association between *SALL4* copy number gain and *SALL4* mRNA expression (*R* = 0.3615, *P* < 0.001, Figures 4D,E) was noted in the TCGA dataset. These findings demonstrate that the DNA copy number gain contributes to the up-regulation of *SALL4* in GC.

### *SALL4*-Induced Key Signaling Cascades and Genes in GC

**Figure 5:**
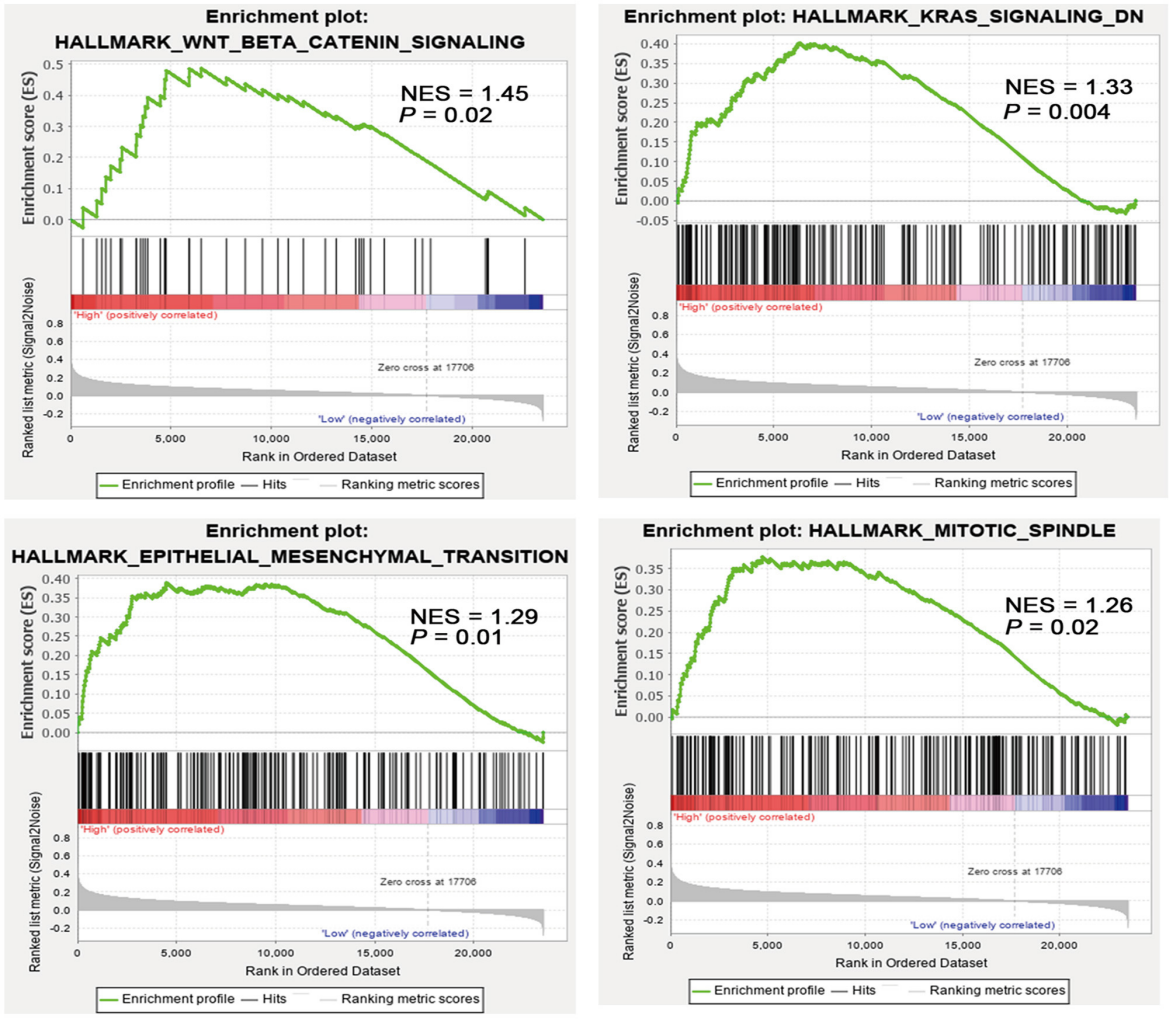
High *SALL4* expression promotes tumorigenesis through multiple pathways. Gene set enrichment analysis (GSEA) of gastric cancer (GC) samples from four GEO datasets (GSE15459, GSE34942, GSE57303, and GSE62254) showed that *SALL4* expression is positively correlated with Wnt/β-catenin signaling, KRAS signaling, mitotic spindle, and epithelial–mesenchymal transition. Each bar corresponds to one gene.

To understand the molecular mechanisms underlying the pro-tumorigenic action of highly expressed *SALL4*, GESA pathway enrichment analysis was performed based on GEO (GSE15459, GSE34942, GSE57303, and GSE62254) and TCGA datasets. The results showed that *SALL4* significantly dysregulated Wnt/β-catenin signaling, KRAS signaling, epithelial–mesenchymal transition and mitotic spindle (Figure 5) in GEO datasets. GSEA results also revealed Wnt signaling, transforming growth factor-beta (TGF-beta) signaling pathways, and gap junction signaling differentially enriched in high *SALL4* expression GC in TCGA dataset (Supplementary Figure 4). We herein assumed that up-regulated *SALL4* also contributed to the poor prognosis of GC *via* the Wnt/β-catenin signaling pathway.

**Figure 6:**
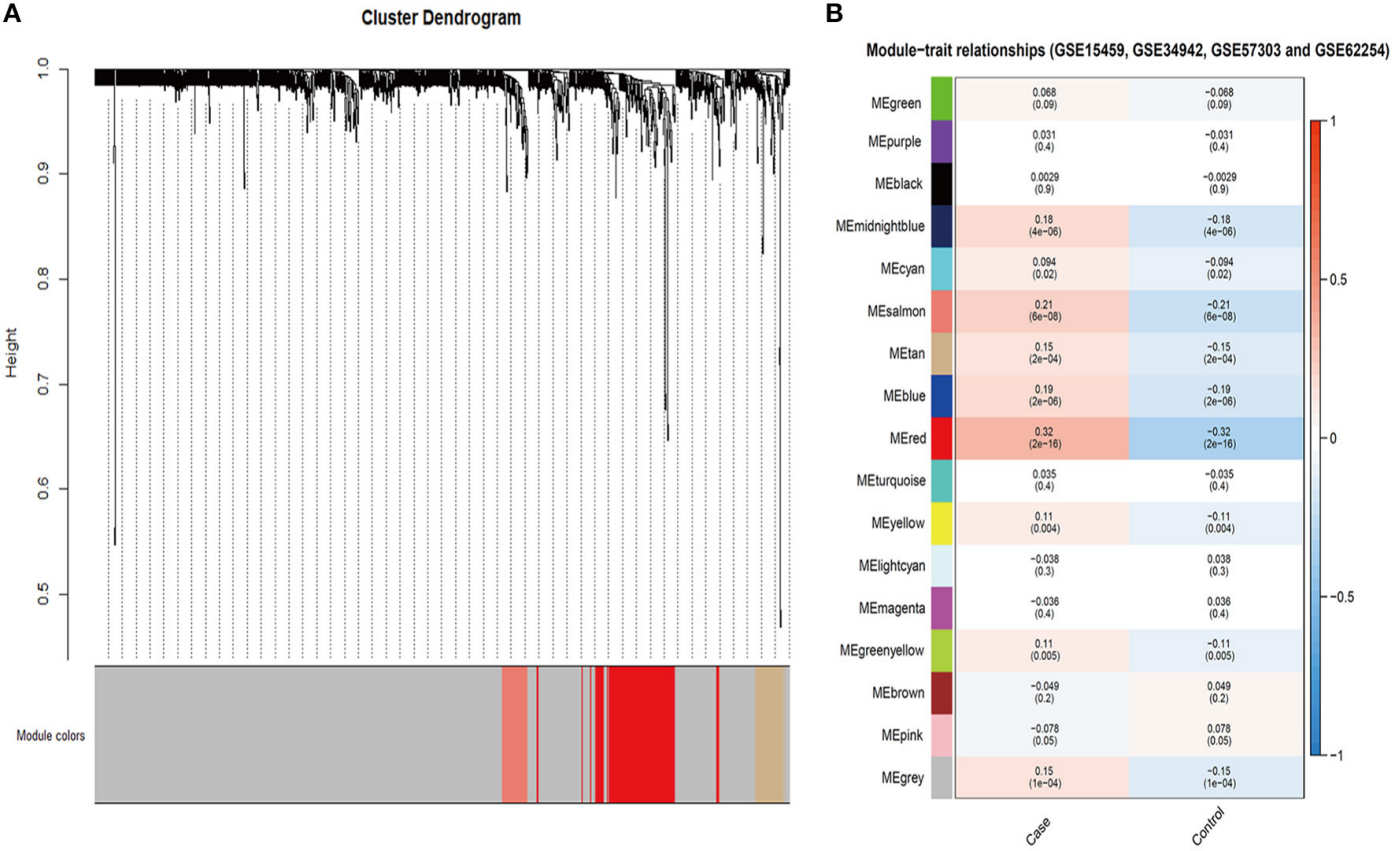
Identification of *SALL4*-related gene modules. Gene modules identified by WGCNA in GSE15459, GSE34942, GSE57303, and GSE62254 **(A)**. The correlation between gene module and trait in four GEO datasets **(B)**. The case represented high *SALL4* expression and control represented low.

**Figure 7:**
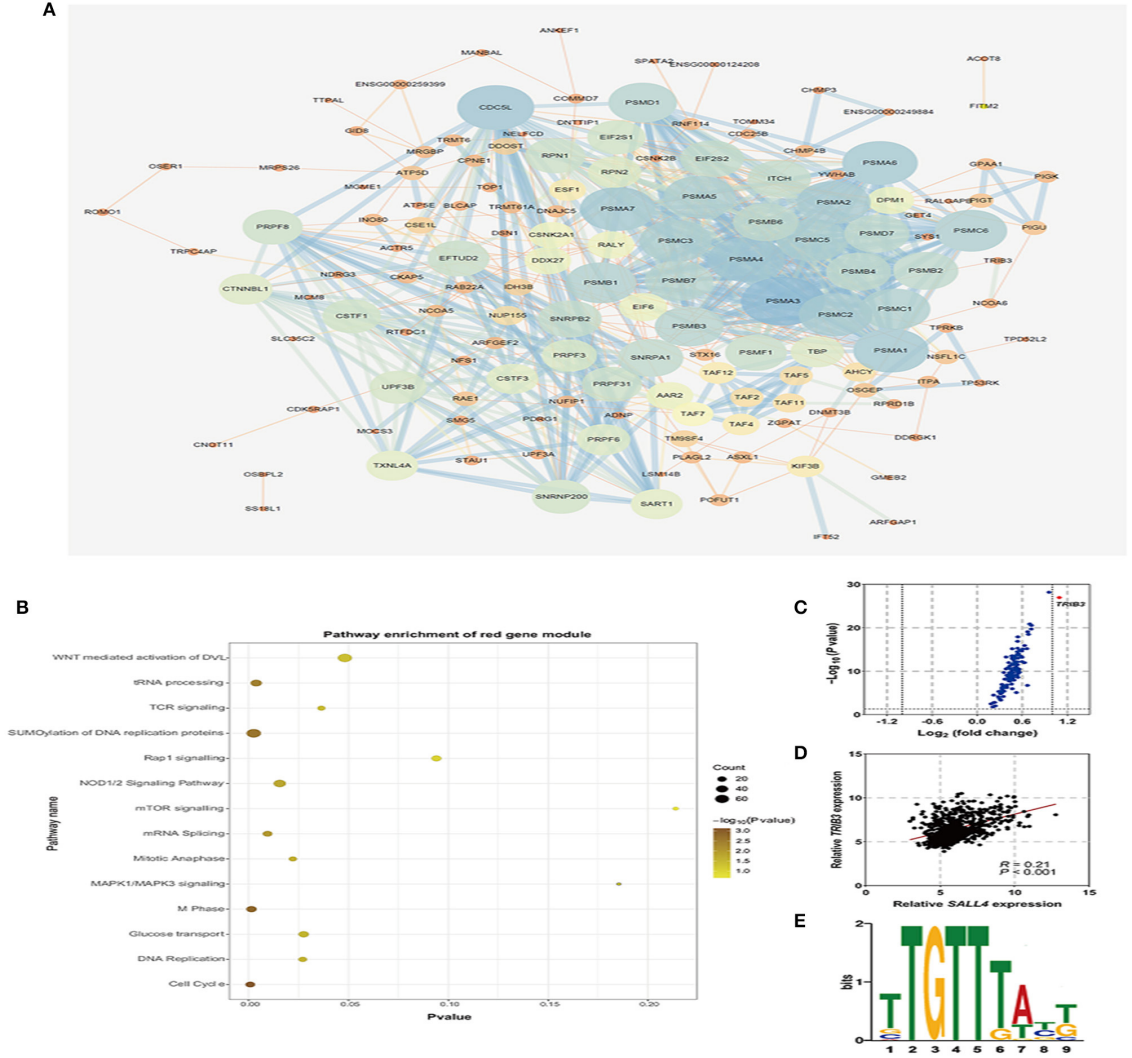
Co-expression genes of SALL4 and its function. Protein–Protein Interaction (PPI) network of the red module, which is the most related gene module of high *SALL4* expression **(A)**. Pathway enrichment analysis of genes in the red module in the PPI network **(B)**. The volcano plot of genes in the red module by the R package limma **(C)**. Scatter plot analysis of mRNA expression of *SALL4* and its co-expression gene *TRIB3* **(D)**. SALL4 binding motif, TTGTTTA(T)T(G)T, was identified by motif analysis tool, MEME-Chip **(E)**.

To further explore the role of *SALL4* in Wnt signaling pathway, we conducted an integrated analysis of four GEO datasets to find *SALL4*-related genes and their function (Figures 6A,B). By using R package WGCNA, we obtain *SALL4*-related genes that include 16 gene modules. The correlation between the trait and gene modules showed that eight gene modules were related with high *SALL4* expression (*P* < 0.05), especially the red module. The genes in the red module were used to construct the PPI network (Figure 7A). To better understand the biological characteristics of the *SALL4*-related gene module, we performed pathway analysis using the genes in the red module by Enrichr. The red module genes were enriched in Wnt-mediated activation of DVL (Figure 7B).

To find the core gene in the red module, we then analyzed the differential expression of genes (DEGs), and *TRIB3* was significantly differentially expressed (Figure 7C). Also, we found that the expression of *SALL4* was positively correlated with that of *TRIB3* (Figure 7D). TRIB3 interacted with the β-catenin–TCF4 complex, thus activating Wnt/β-catenin signaling (Zhang et al., 2019), pointing that activation of Wnt signaling by *SALL4* may need *TRIB3*. It was evident that SALL4, as a transcriptional regulator, trans-activates *CTNNB1* by binding to the promoter of *CTNNB1* (Chen et al., 2019), and we wondered whether *SALL4* activates Wnt signaling through regulating *TRIB3* at the transcriptional level. Using an online motif analysis tool, MEME-Chip, to screen SALL4 binding motifs, one binding motif has been identified: TTGTTTA(T)T(G)T (Figure 7E). Collectively, the present results suggested that the up-regulation of SALL4 may activate the oncogenic Wnt signaling pathway to promote gastric tumorigenesis by influencing the transcriptional mechanisms of *TRIB3*.

## Discussion

The clinical outcomes of GC differ among patients, and TNM staging is used as a conventional clinical prognostic indicator that only partially reflects biological malignancy. Therefore, prognostic biomarkers of oncogenic potential are required for better risk assessment. This study aimed to investigate whether *SALL4*, a putative oncofetal gene, influences the prognosis of GC patients, and to investigate the mechanism underlying its effects.

Although *SALL4* has been reported as a prognosis-related biomarker for many cancers, such as liver cancer, esophageal cancer, germ cell cancer, and breast cancer (Cao et al., 2009a; Liu T.-C. et al., 2014; Yue et al., 2015; He et al., 2016), the correlation between *SALL4* expression and GC prognosis has not been clarified. Previous studies on the prognostic significance of *SALL4* in GC are limited by their small sample size (103 cases), and the clinical significance of *SALL4* has been evaluated exclusively at the mRNA level, rather than at the protein level (Liu J. et al., 2014). This study, which included a larger series of 1,815 patients, reported that 16.7% of GC tumors were SALL4-positive and that SALL4 positivity was associated with cancer progression-related clinicopathologic parameters, such as advanced stage, lymph node metastasis, and vascular invasion. Our findings are consistent with those of previous reports, which indicated that SALL4 levels were positively associated with lymph node metastasis and that SALL4 is an indicator of metastatic potential in GC (Zhang et al., 2018). A novel finding of our study is that SALL4 is negatively associated with EBV infection in GC. EBVaGC, a molecular subtype classified by TCGA that accounts for approximately 5% of GC cases, has distinct clinicopathological and genetic features, and EBV infection and a lack of SALL4 may mark a group of GC patients with a better prognosis (Yang et al., 2019).

In both univariate and multivariate Cox regression analyses, we noted that SALL4-positive was associated with poor survival in GC. Notably, among patients with stage I and II GC, a SALL4-positive status has a poorer overall survival than a SALL4-negative status. There was no prognostic value among stage III GC patients, implying that SALL4 may predict aggressive types in the early stages of GC. We compared the present results with data from the TCGA STAD dataset and four Asian GC cohorts from GEO by analyzing the association between *SALL4* mRNA expression and patient prognosis.

Previous studies have demonstrated the regulation of *SALL4* overexpression in cancer. Hypomethylation of the promoter region of *SALL4* has been observed in myelodysplastic syndrome (MDS) and acute myeloid leukemia (AML) and is strongly associated with high mRNA levels of *SALL4* (Lin et al., 2013; Ma et al., 2013). Also, some studies have demonstrated that miR-16 and lncRNA DANCR mediate the up-regulation of SALL4 (Zhou et al., 2016; Pan et al., 2018). *SALL4* is reportedly up-regulated in CDX1-positive intestinal metaplasia of the stomach in both humans and mice (Bard et al., 2009; Fujii et al., 2012). The up-regulated group plays an important role in tumor stemness, drug resistance, apoptosis, cell proliferation, and invasion, highlighting the need to further the current understanding of *SALL4* regulation in GC. Several studies on GC have recently suggested gene amplification to be associated with up-regulation (Gorringe et al., 2005; Tsukamoto et al., 2008). In this study, we also found a positive correlation between CN vs. and the mRNA expression of *SALL4* in the TCGA dataset, suggesting that the *SALL4* copy number gain may contribute to its overexpression in GC.

Several lines of evidence suggest that overexpression of *SALL4* in human cancers affects multiple cellular processes involved in tumorigenesis, tumor growth, and tumor progression (Ma et al., 2006; Yang et al., 2008; Itou et al., 2013). To explore how *SALL4* affects tumor behavior, especially the molecular mechanisms relevant to *SALL4* in GC, we identified the relevant pathways of *SALL4* and its co-expressed genes by pathway enrichment analysis and WGCNA. By comparing the enrichment of DEGs with *SALL4* in GC, we demonstrated that high expression of *SALL4* GC was more concentrated in several signaling pathways, such as Wnt/β-catenin, epithelial–mesenchymal transition, KRAS, and TGF-beta signaling pathways. Several studies have shown that the deregulation of *CD44* and TGF-beta signaling could be involved in the *SALL4*-mediated oncogenic mechanisms (Yuan et al., 2016; Zhang et al., 2018). *SALL4* expression has also been reported to be regulated by the Wnt signaling pathway in cervical cancer, hepatocellular carcinoma, and esophageal squamous cell carcinoma (Böhm et al., 2006; He et al., 2016). The Wnt signaling pathway is strongly induced in tumorigenesis, activating kinases to transmit extracellular signals that regulate cell growth, differentiation, proliferation, apoptosis, and migration. Meanwhile, the results of correlation analyses indicated that the co-expression genes of *SALL4*, such as *TRIB3*, exhibited positive correlations with *SALL4* expression. *TRIB3* is a core gene in the Wnt signaling pathway by interacting with β-catenin. A previous study demonstrated that *SALL4* could bind to the promoter of *CTNNB1* (the gene name of β-catenin) and further activate Wnt/β-catenin signaling in cervical cancer cells, and then we explored whether the transcriptional regulator *SALL4* could regulate the expression of *TRIB3*. Using MEME-Chip, we screen the putative SALL4 binding motif: TTGTTTA(T)T(G)T that is found in the promoter region of *TRIB3*. Together, activation of the Wnt pathway and *SALL4*-related genes may result in poor survival in GC, giving new ideas for the follow-up study.

In conclusion, an analysis of 1,815 consecutive GC cases revealed that patients with SALL4-positive GC experience significantly worse outcomes than SALL4-negative patients, indicating that SALL4 is a prognostic indicator in GC. The corresponding increase in the regional copy number of *SALL4* and *SALL4* mRNA overexpression suggests that its overexpression may due to DNA copy number changes. Besides, *SALL4*, as well as its co-expressed genes, could potentially activate several pathways, especially the Wnt signaling pathway, which is closely associated with a worse prognosis in GC patients.

## Data Availability Statement

The datasets presented in this study can be found in online repositories. The names of the repository/repositories and accession number(s) can be found in the article/Supplementary Material.

## Ethics Statement

The studies involving human participants were reviewed and approved by the Ethics Committee of Peking University Cancer Hospital. The patients/participants provided their written informed consent to participate in this study. Written informed consent was obtained from the individual(s) for the publication of any potentially identifiable images or data included in this article.

## Author Contributions

LZ and JJ were the principal investigator and responsible for the study design. YY, ZiL, ZB, and XWu collected and assembled data. XWa and YY performed data analysis, interpretation, and drafted the manuscript. YH provided gastric tissues from biobank. YL and ZhL contributed to pathologist scoring. All authors have read and approved the final version of the manuscript.

## Conflict of Interest

The authors declare that the research was conducted in the absence of any commercial or financial relationships that could be construed as a potential conflict of interest.
