# Supplementary material for: Up-Regulation of SALL4 Is Associated With Survival and Progression via Putative WNT Pathway in Gastric Cancer
Source: Front Cell Dev Biol. 2021 Feb 11;9:600344. doi: 10.3389/fcell.2021.600344 (PMC7905055; doi:10.3389/fcell.2021.600344)
Supplement: Supplementary file 1 [file Table_1.DOCX]

Supplementary Material

# Supplementary Figures and Tables

## Supplementary Figures


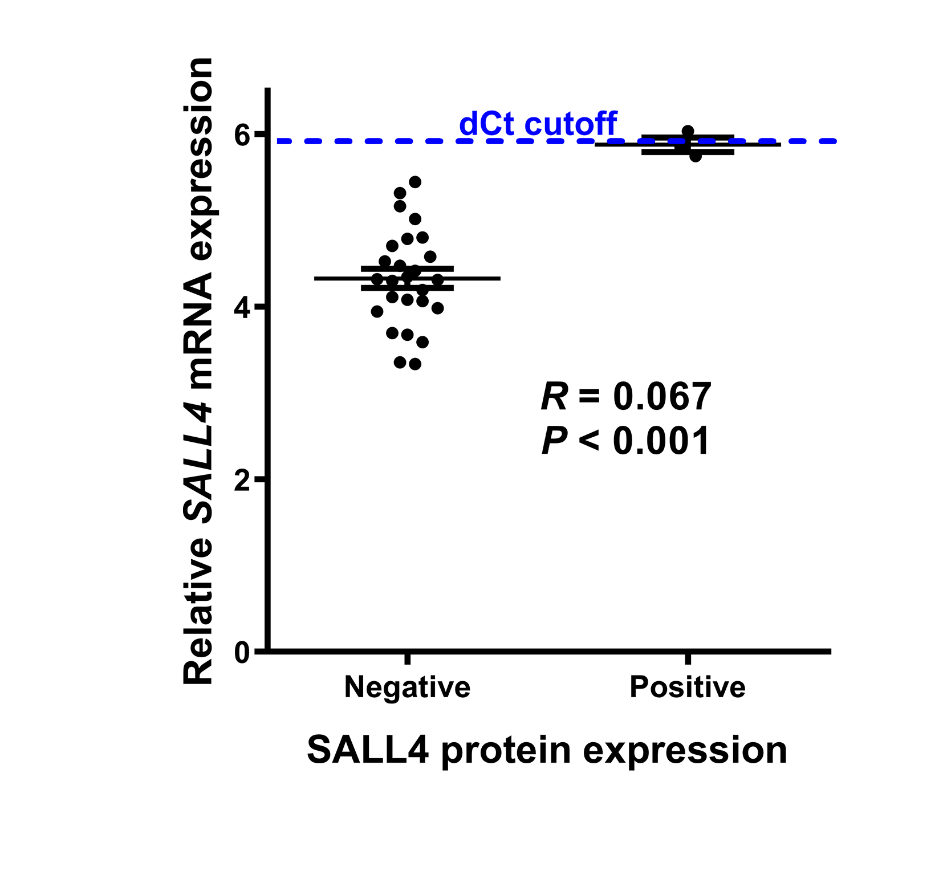


**Supplementary Figure 1.** Graph of SALL4 dCt values by SALL4 IHC result categorized as negative and positive. Comparison of SALL4 status determined by IHC and RT-qPCR.


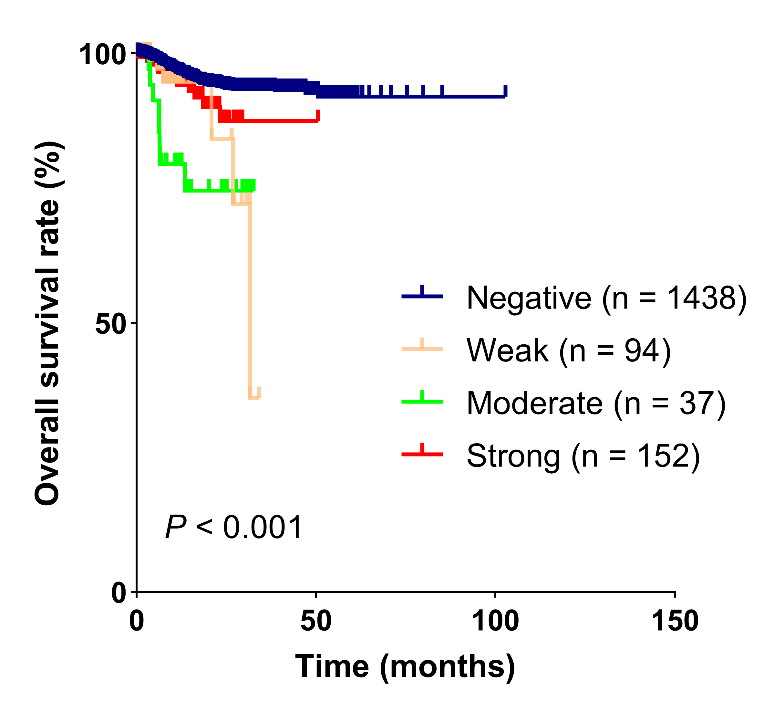


Supplementary Figure 2. Kaplan-Meier survival curves for all patients with SALL4 negative, weak, moderate, and strong staining.

**
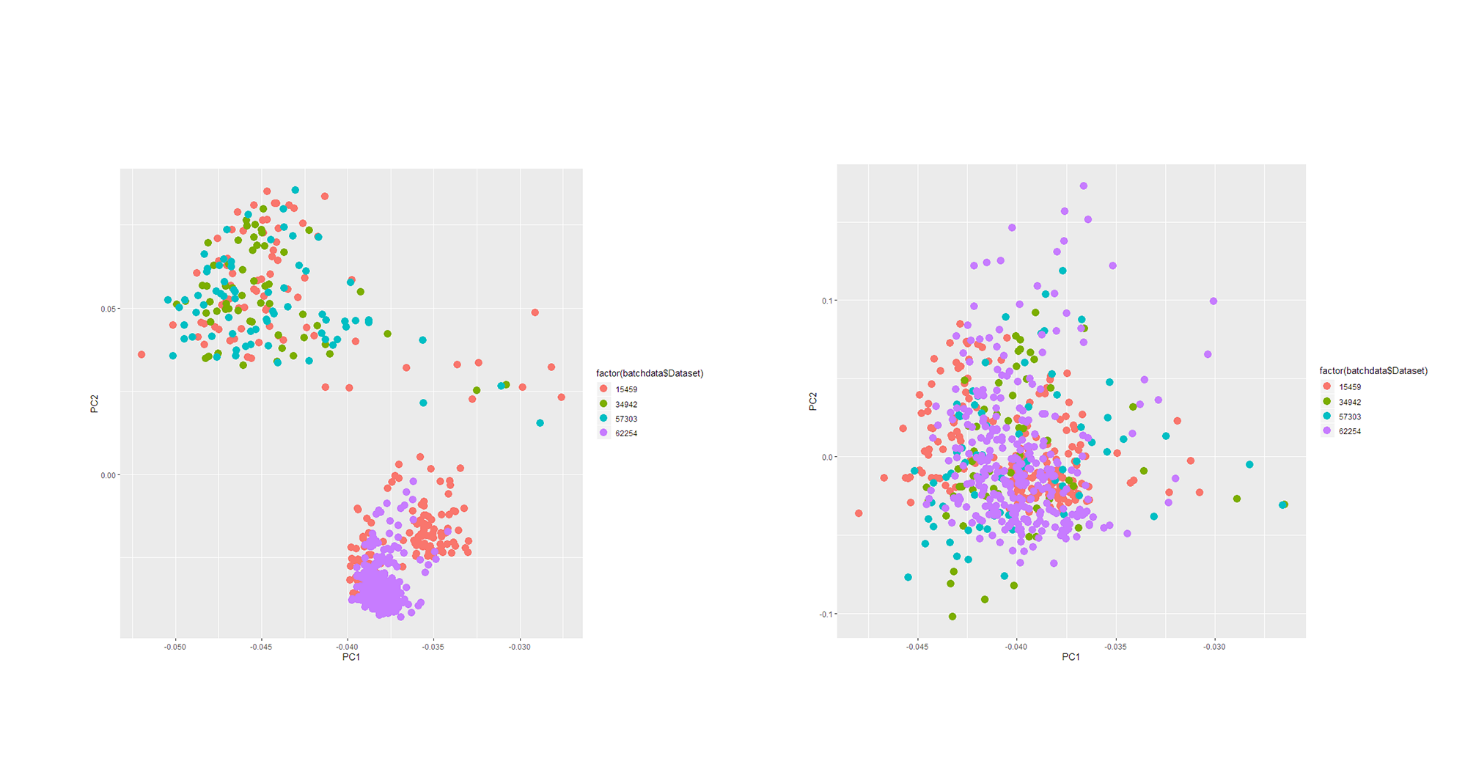
**

Supplementary Figure 3. Scatter plot depicting principal component analysis of genes across the integrated 4 GEO cohorts (GSE15459, GSE34942, GSE57303, GSE62254) before (left) and after (right) batch correction.

**
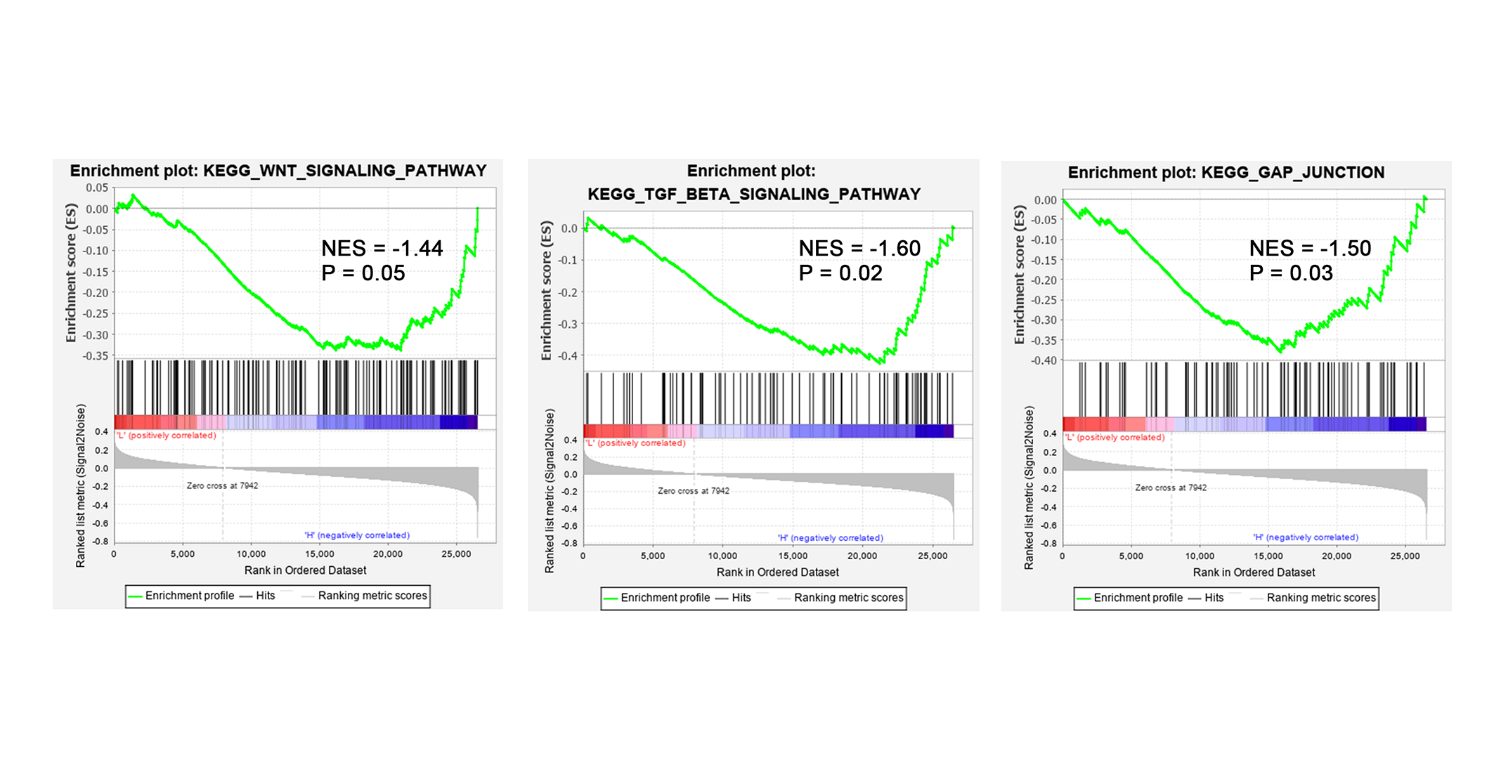
**

Supplementary Figure 4. Gene set enrichment analysis (GSEA) of gastric cancer (GC) samples from TCGA dataset.


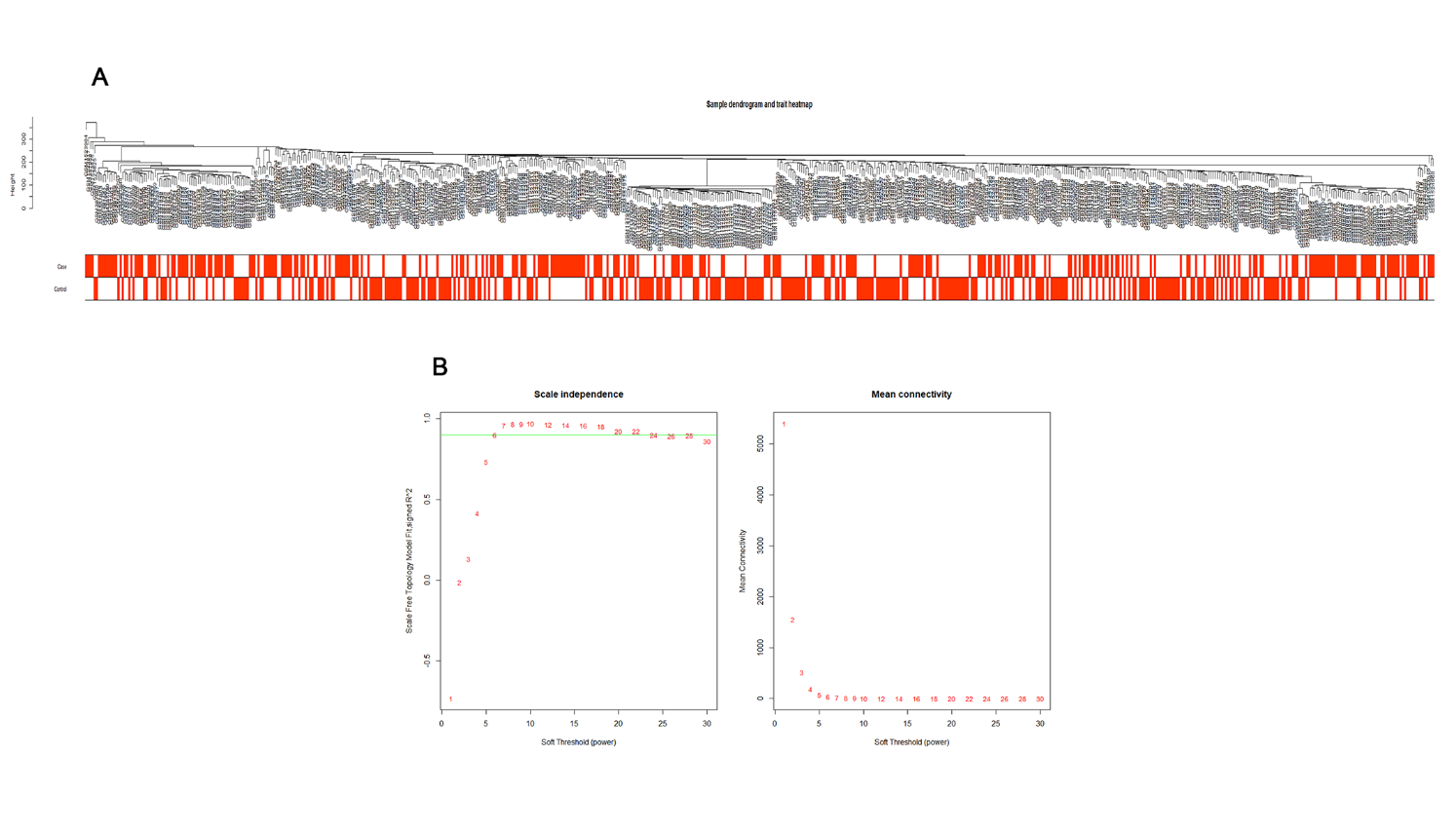


Supplementary Figure 5. Hierarchical clustering of samples in GSE15459, GSE34942, GSE57303 and GSE62254 (A). Determination of soft-thresholding power in WGCNA (B).


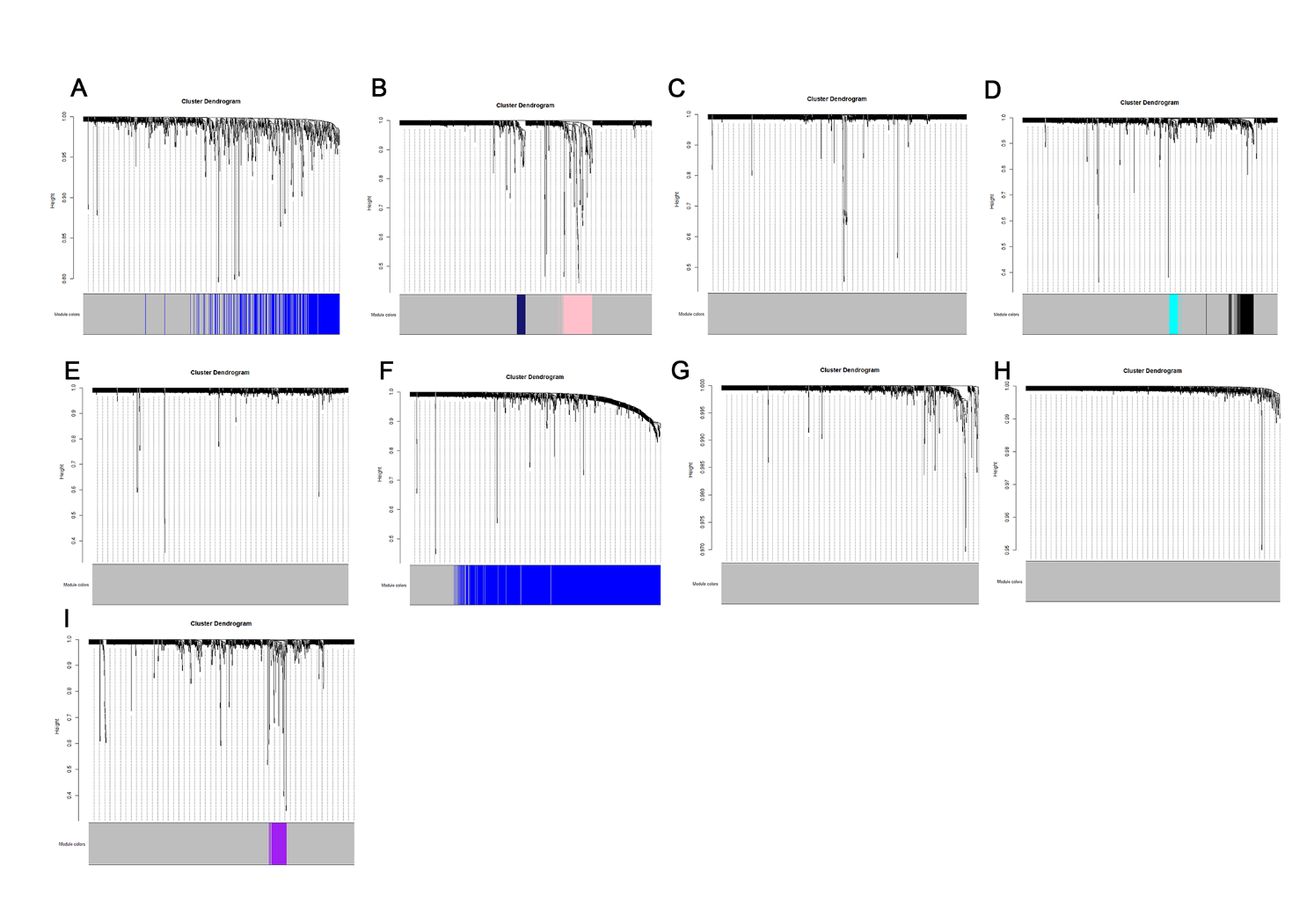


Supplementary Figure 6. Clustering dendrograms of identified *SALL4*-related genes modules in GSE15459, GSE34942, GSE57303 and GSE62254.


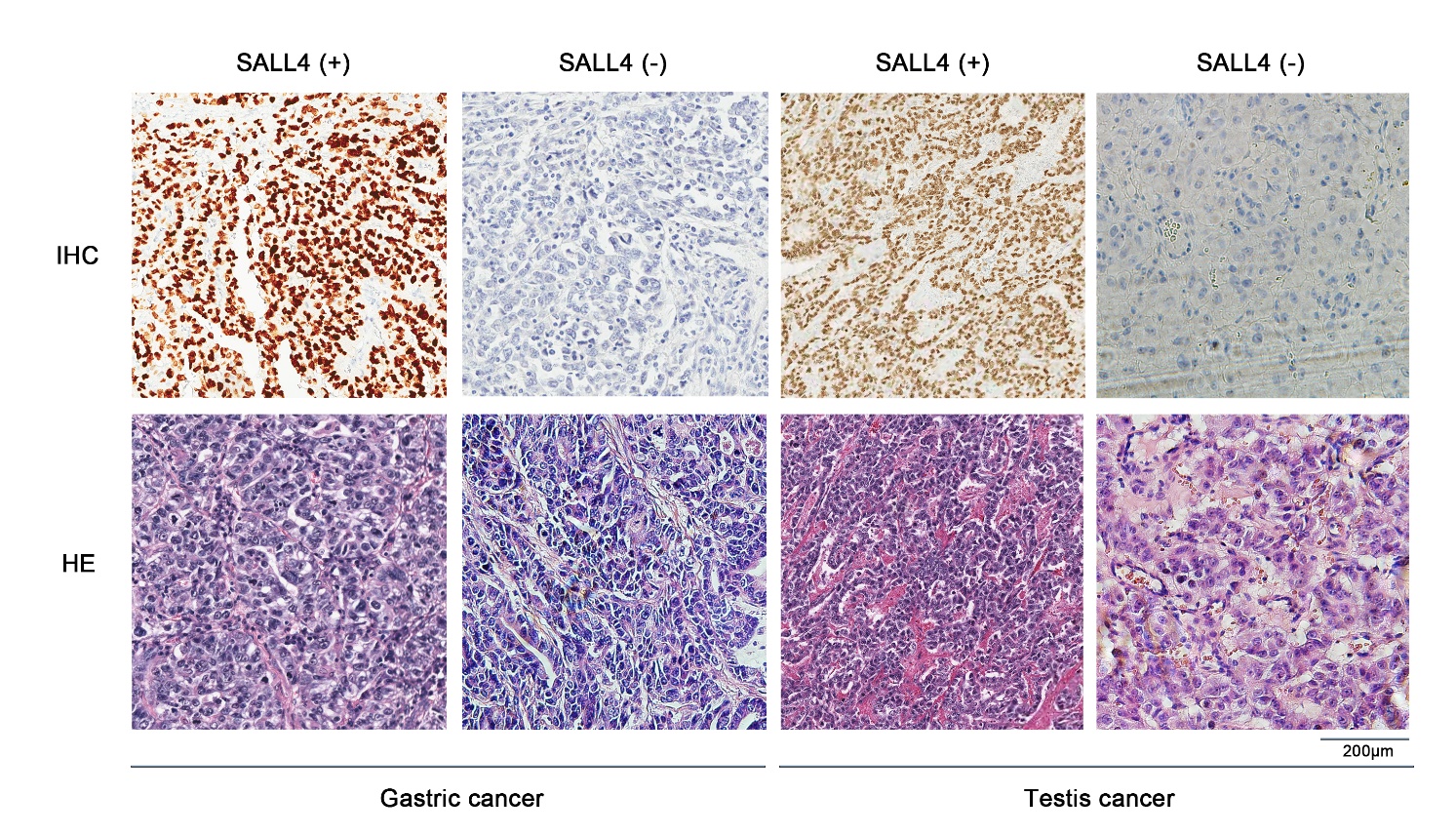


Supplementary Figure 7. SALL4 expression in human primary gastric cancer and testis cancer by immunohistochemistry (IHC) and hematoxylin-eosin staining (HE).

Supplementary Table 1. Baseline information of gastric cancer in our study

| Variables | N |
| --- | --- |
| Sex |  |
| Male | 1296 (71.4%) |
| Female | 519 (28.6%) |
| Age (years) |  |
| 18–55 | 646 (35.6%) |
| 56–90 | 1169 (64.4%) |
| Differentiation* |  |
| Poorly | 795 (43.8%) |
| Moderately | 962 (53.0%) |
| Well | 53 (2.9%) |
| WHO classification |  |
| Adenocarcinoma | 1795 (98.9%) |
| Non-adenocarcinoma | 20 (1.1%) |
| Lauren |  |
| Diffused-type | 505 (27.8%) |
| Intestinal-type | 694 (38.3%) |
| Mixed-type | 616 (33.9%) |
| Location |  |
| Cardia | 443 (24.4%) |
| Non-cardia | 1372 (75.6%) |
| TNM stage |  |
| Stage Ⅰ | 502 (27.7%) |
| Stage Ⅱ | 538 (29.6%) |
| Stage Ⅲ | 761 (41.9%) |
| Stage Ⅳ | 14 (0.8%) |
| Lymphatic metastasis* |  |
| Positive | 1072 (59.1%) |
| Negative | 740 (40.8%) |
| Distant metastasis |  |
| Positive | 14 (0.8%) |
| Negative | 1801 (99.2%) |
| Vascular invasion* |  |
| Positive | 974 (53.7%) |
| Negative | 837 (46.1%) |
| Perineural invasion* |  |
| Positive | 863 (47.5%) |
| Negative | 944 (52%) |
| SALL4 |  |
| Positive | 304 (16.7%) |
| Negative | 1511 (83.3%) |

*Data for differentiation, lymph node metastasis, vascular invasion, perineural invasion were missing for 5, 3, 4, 8 patients respectively; WHO, World Health Organization

Supplementary Table 2. Baseline information of gastric cancer in TCGA database

| Variables | N |
| --- | --- |
| Sex |  |
| Male | 216 (64.7%) |
| Female | 118 (35.3%) |
| Age (years) |  |
| 18–55 | 59 (17.7%) |
| 56–90 | 275 (82.3%) |
| TNM stage* |  |
| Stage I | 44 (13.2%) |
| Stage Ⅱ | 106 (31.7%) |
| Stage Ⅲ | 137 (41.0%) |
| Stage Ⅳ | 33 (9.9%) |
| Lymphatic metastasis* |  |
| Positive | 225 (67.4%) |
| Negative | 98 (29.3%) |
| Distant metastasis* |  |
| Positive | 22 (6.6%) |
| Negative | 300 (89.8%) |
| *SALL4* |  |
| High | 240 (71.9%) |
| Low | 94 (28.1%) |

*Data for stage, lymph node metastasis, distant metastasis and SALL4 were missing for 14, 11, 12 patients respectively.
